# Altered Rest-Activity Patterns Evolve via Circadian Independent Mechanisms in Cave Adapted Balitorid Loaches

**DOI:** 10.1371/journal.pone.0030868

**Published:** 2012-02-13

**Authors:** Erik R. Duboué, Richard L. Borowsky

**Affiliations:** Department of Biology, New York University, New York, New York, United States of America; Yale School of Medicine, United States of America

## Abstract

Circadian rhythms and rest homeostasis are independent processes, each regulating important components of rest-activity patterns. Evolutionarily, the two are distinct from one another; total rest time is maintained unaffected even when circadian pacemaker cells are ablated. Throughout the animal kingdom, there exists a huge variation in rest-activity patterns, yet it is unclear how these behaviors have evolved. Here we show that four species of balitorid cavefish have greatly reduced rest times in comparison to rest times of their surface relatives. All four cave species retained biological rhythmicity, and in three of the four there is a pronounced 24-hour rhythm; in the fourth there is an altered rhythmicity of 38–40 hours. Thus, consistent changes in total rest have evolved in these species independent of circadian rhythmicity. Taken together, our data suggest that consistent reduction in total rest times were accomplished evolutionarily through alterations in rest homeostasis.

## Introduction

Circadian rhythms, defined as synchronized, free-running, oscillations in biological processes with a period length close to 24 hours, regulate a number of biological phenomena including gene expression, feeding, and locomotor activity. Circadian rhythms can be entrained by any number of cues, including the light-dark cycle, temperature fluctuations and feeding cycles. These rhythmic behaviors are important to the survival of an organism and have been conserved from organisms as primitive as bacteria [1] and fungi [2] through plants, insects [3] and vertebrates [4].

Particular attention in recent years has been focused on circadian locomotor activity with respect to the evolution of rest-activity (RA) patterns and sleep [5]. Sleep can be defined as a homeostatically regulated process marked behaviorally by reduced responsiveness to external stimuli, which occur in a circadian manner. Specifically, RA patterns and sleep are thought to have evolved from a more ancestral circadian locomotor activity. In higher order species such as insects and vertebrates, RA patterns are thought to be regulated by the interaction of two main processes, namely Process-S, which regulates the homeostatic component, and process-C, which regulates the circadian component [5]. The theory that these RA patterns have diverged into distinct, independent processes is supported by the observation that total sleep time and the homeostatic response to sleep deprivation persist when the vertebrate circadian pacemaker, the Suprachiasmatic Nucleus, is ablated [6]. Thus, even in the absence of circadian rhythms, sleep homeostasis persists.

There is enormous variation in RA patterns throughout the animal kingdom [7], [8]. While allelic variations in core clock genes can lead to altered total rest times [9], there are a number of well documented non-clock genes such as the hypocretin 2 receptor [10] that directly alter RA patterns without affecting circadian rhythms [11]. Thus, it is not clear if RA patterns evolve as a consequence of altered circadian rhythms, or if the genetic mechanisms leading to changes in RA patterns in natural populations evolved independently and are primarily a consequence of reduction in the homeostatic need.

Many cave organisms have converged on a suite of phenotypic traits including reduced optic and enhanced extra-optic senses, and depigmentation making cave organisms particularly suited to the study of evolutionary phenomena [12]. The ecology of cave and surface environments differ in several important ways; caves are without light and photosynthesis, generally have reduced food availability, and typically are more stable than surface environments in terms of temperature variability [13]. Thus, the cave environment offers its inhabitants fewer zietgeber cues than are provided to surface animals. Furthermore, recent studies have pointed to both altered circadian rhythms [14] and sleep loss [15] in hypogean morphs. These organisms therefore provide a powerful model for evolutionary study of both circadian rhythms and RA patterns.

We studied circadian rhythms and RA patterns in several species of the SE Asian hillstream loaches (Family Balitoridae) to specifically address these questions. Balitorids are widespread throughout surface streams of the Old World and in certain regions, most prominently in SE Asia and Southern China, have given rise to numerous cave adapted species.

## Results

**Figure 1:**
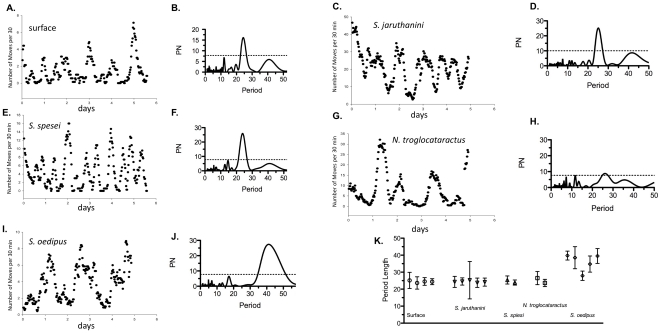
Both cave and surface balitorid species show pronounced locomotor rhythms. Activity was measured as the number of movements per 30 min period (y-axis) and was plotted over the full period of observation. Individual actogram plots and LSP periodograms for surface (A, B), *S. jaruthanini* (C,D), *S. spesei* (E.,F), *N. troglocataractus* (G, H) and *S. oedipus* (I, J) over the full observation period show pronounced rhythmicity in all species. Period length as measured by the Lomb-Scargle algorithm (K) reveal a 24-hour circadian rhythm in all but one cave species. Period values in K represent peak period length (h)±1 LOD.

We first asked if surface and cave balitorids exhibited a circadian rhythm. To assess circadian periodicity in balitorid populations, we recorded locomotor activity for several days in constant dark (DD) conditions, as is standard for measuring free running rhythms. Surface fish populations exhibited an average 24-hour circadian rhythm (Figure 1A, 1B, 1K; 24.19±0.19 hr). We compared these data to those of cave dwelling balitorids and found significant differences between groups (Kruskal Wallis H_4,18_ = 10.38; p<0.03). Interestingly, we found that all but one cave population studied exhibited approximate 24-hour (circadian) rhythms that were not significantly different that those of the surface form (*N. troglocataractus* showed weak, but significant rhythms as measured by Lomb-Scargle). *S. oedipus*, however, showed a significantly longer rhythm than both the surface and other cave populations (*S. jaruthanini*: 23.19±3.11 hr.; *S. spiesi*: 24.06±0.19 hr.; *N. troglocataractus*: 24.92±1.03 hr.; *S. oedipus*: 38.50±1.25 hr., p<0.01; Figure 1C–K). These results suggest that of the balitorid species studied, while all populations showed robust rhythms, only one population diverged from an approximate 24 hr. (circadian) rhythm to a 38 hr. rhythm (Figure 1K).

**Figure 2:**
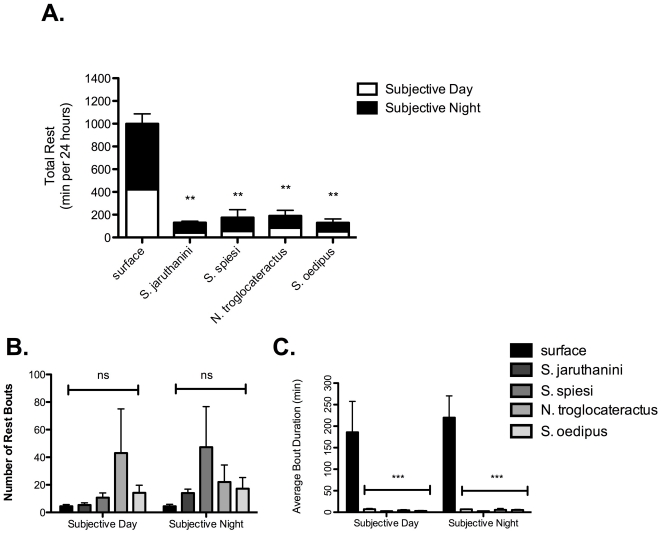
Reduced total rest time in cave balitorids is primarily a function of reduced rest bout duration. Quantification of total rest (A.) indicates that surface fish spend an average of 999.1±64.4 min per 24 hr in a rest-like state. In strong contrast to the surface dwelling form, average total rest per 24-hour period was significantly less in all four of the cave species studied (*S. oedipus*: 128.8±62.04 min, p<0.001; *S. jaruthanini*: 130.4±27.38 min, p<0.001; *N. troglocataractus*: 202.3±122.1 min, p<0.001; *S. speisi*: 161.0±72.51 min, p<0.001). (B–C.) There were no significant differences between the number of rest bouts in surface and cave balitorids (B. Day - Epigean: 4.4±1.3; S. oedipus: 14.2±5.5; S. jaruthanini: 5.4±1.5; N. *troglocataractus*: 43.0±32.1; S. speisi: 10.7±3.5; Night - Epigean: 4.4±1.4; S. oedipus: 17.1±8.2; S. jaruthanini: 14.0±2.8; N. *troglocataractus*: 22.0±12.3; S. speisi: 47.3±29.3) thought the duration of each rest bout was significantly less in all cave populations studied compared to surface (Day - Epigean: 185.8±71.7; *S. oedipus*: 13.2±0.9, p<0.001; *S. jaruthanini*: 16.8±1.8, p<0.001; *N. troglocataractus*: 14.4±1.4, p<0.001; *S. speisi*: 12.7±0.6, p<0.001; Night - Epigean: 219.8±50.7; S. oedipus: 15.2±1.3, p<0.001; *S. jaruthanini*: 16.7±0.7, p<0.001; *N. troglocataractus*: 15.4±3.1, p<0.001; *S. speisi*: 12.5±0.1, p<0.001). For panel A., white denotes subjective day (CT0–CT12) and black denoted subjective night (CT12–CT24). For panels B. and C., Black denotes surface, dark-shade grey denotes *S. jaruthanini*, mid-shade grey denotes *S. spesei*, light-shade grey denotes *N. troglocataractus* and white denotes *S. oedipus*. All plots represent mean ± standard error of the mean (SEM). Asterisks represent significance relative to surface fish.

We next asked if there was a change in RA patterns between populations. Because we wished to compare rest-activity results to circadian results, we initially quantified rest-activity patters in individuals recorded under D∶D conditions, as presented in our circadian studies. Quantification of total rest revealed significant differences among groups (Kruskal Wallis H_4,23_ = 16.22; p<0.01). The surface species had an average rest time of 999.1±64.4 min per 24-hour period. In strong contrast to the surface dwelling form, average total rest per 24-hour period was significantly less in all four of the cave species studied (*S. oedipus*: 128.8±62.04 min, p<0.001; *S. jaruthanini*: 130.4±27.38 min, p<0.001; *N. troglocataractus*: 202.3±122.1 min, p<0.001; *S. speisi*: 161.0±72.51 min, p<0.001; Figure 2A).

A lack of circadian rhythms does not always lead to a change in total rest over a given 24-hour period, but may manifest itself as changes in the distribution of total rest between the day and night phases [6]. Therefore, we looked at differences in day and night (i.e., subjective day and night, herein) rest across balitorid species. Consistent with our circadian data, we noticed that for all species studied, average rest was greater during periods of night compared to day (Figure 2A). Quantification of rest during day and night also revealed significant differences among groups (day: Kruskal Wallis H_4,23_ = 14.96; p<0.01, night: Kruskal Wallis H_4,23_ = 15.91; p<0.01). Epigean balitorids exhibited day and night rest times of 422.0±62.09 min and 577.0±87.9 min, respectively. Compared to the surface balitorids, all four of the cave populations studied showed significant differences in both average day rest (*S. oedipus*: 52.0±28.8 min, p<0.001; *S. jaruthanini*: 41.8±15.89 min, p<0.001; *N. troglocataractus*: 84.0±51.12 min, p<0.001; *S. speisi*: 55.67±26.69 min, p<0.001) and night rest (*S. oedipus*: 76.8±34.04 min, p<0.001; *S. jaruthanini*: 88.6±13.07 min, p<0.001; N. *troglocataractus*: 118.3±70.93 min, p<0.001; S. speisi: 105.3±49.6 min, p<0.001) suggesting that in these individuals, adaptation to a cave environment is accompanied by significant reductions in total rest (Figure 2A).

Total rest is a function of two main components, the number of rest bouts exhibited by an individual and the average duration of each bout. We quantified both the numbers and average durations of both subjective day and night rest bouts for all species (Figure 2B and 2C). Neither daytime bout numbers (Epigean: 4.4±1.3; S. oedipus: 14.2±5.5; S. jaruthanini: 5.4±1.5; N. *troglocataractus*: 43.0±32.1; S. speisi: 10.7±3.5) nor nighttime bout numbers (Epigean: 4.4±1.4; S. oedipus: 17.1±8.2; S. jaruthanini: 14.0±2.8; N. *troglocataractus*: 22.0±12.3; S. speisi: 47.3±29.3) were significantly different between any of the cave species and the surface forms (Figure 2B). Bout durations however, were significantly different between surface and all cave species during both the day and night phases (day: Kruskal Wallis H_4,23_ = 15.17; p<.01; night: Kruskal Wallis H_4,23_ = 15.369; p<0.01; Figure 2C). The epigean form exhibited daytime and nighttime bout durations of 185.8±71.7 min and 219.8±50.7 min, respectively. All four hypogean forms exhibited significantly shorter rest duration periods for both day (*S. oedipus*: 13.2±0.9, p<0.001; *S. jaruthanini*: 16.8±1.8, p<0.001; *N. troglocataractus*: 14.4±1.4, p<0.001; *S. speisi*: 12.7±0.6, p<0.001) and night (S. oedipus: 15.2±1.3, p<0.001; *S. jaruthanini*: 16.7±0.7, p<0.001; *N. troglocataractus*: 15.4±3.1, p<0.001; *S. speisi*: 12.5±0.1, p<0.001) phases.

Because RA patterns are often studied in L∶D cycles and constant dark conditions may have an effect on total rest, we sampled a subset of surface and cave populations and retested rest on an 12∶12 L∶D cycle. Results of these L∶D studies were comparable to those of the D∶D studies. Quantification of total rest again revealed significant differences among groups (Kruskal Wallis H_3, 27_ = 12.25; p<0.01). The surface species had an average rest time of 802.2±143.5 min per 24-hour period. Average total rest per 24-hour period was significantly less in two of the three cave species studied. Both *S. kaysonei* (224.8±86.9 min, p<0.05) and *S. spiesi* (79.0±14.3 min, p<0.01) differed significantly. Average total rest in *S. jaruthanini* was less than in the surface species (279.6±108.1 min), but not significant. These data suggest that reduced rest in cave balatorids is independent of light cues.

## Discussion

Taken together, our data suggest that cave balitorid populations from SE Asia have retained biological rhythms, yet all evolved a phenotype whereby they significantly reduce rest during a 24-hour period. We found significant locomotor rhythms to be present in all species studied. Moreover, we found that 3 of 4 cave populations studied maintained a 24-hour circadian rhythm, suggesting under some but not all conditions, the cave environment may lead to changes in circadian rhythmicity. Our data suggest that while all populations have maintained a biological rhythm, one population in particular had an apparent shift from a 24-hour rhythm to a near 38-hour rhythm. While it is not impossible to rule out the possibility of two rhythms with individual period lengths in *S. oedipus* (one of high amplitude and one of low amplitude) which could skew LSP to an artificially longer period length, it is interesting to note that a shift in rhythmicity from a 24-hour rhythm to one significantly longer has been reported in at least one other cavefish population, the Somalian cavefish, *Phreatichthys andruzzii* [16]. While the evolutionary basis for this apparent shift in periodicity is as yet unclear, the most parsimonious explanation is genetic drift. We believe that our data can be explained by an apparent relaxed selective pressure on a 24-hour rhythm.

In contrast to period length, all cave populations studied showed significant reductions in total rest over a 24 hour period. This convergence on reduced rest may suggest a general adaptation in hypogean fish; we previously reported that three independently derived populations of the Mexican Blind Cavefish, *Astyanax mexicanus*, have all converged on a similar phenotype of reduced sleep in comparison to surface populations [15]. The reduced sleep is primarily a function of reduced bout duration; bout numbers are the same in both surface and cave dwelling *Astyanax*. We now have extended this inquiry to include RA patterns in the geographically and taxonomically distinct balitorids. Absent an exhaustive survey of all behavioral correlates of sleep in the balitorid species studied, we can only refer to the phenomenon demonstrated in the present paper as rest. However, the present results alongside those on *Astyanax* clearly suggest a general convergence on increased activity in cavefish species.

Lastly, we believe this is one of the first studies testing the evolution of rest-activity patterns with respect to the dual process theory. Specifically, because rest-activity patterns (and sleep) are regulated and maintained through both circadian and homeostatic processes, and both of these processes are independent of one another, rest patterns could evolve via allelic variation in genes regulating Process-C, through allelic variation in genes regulating Process-S, or both. Our data show that three of the four cave balitorids we studied exhibited circadian (app. 24 hour periodicity) rhythms, while one species has diverged significantly from a 24-hour to a 38–40 hour periodicity. In strong contrast, all populations showed a significant reduction in total rest time over a 24-hour period. We believe these data suggest that rest activity patterns are much more likely to evolve through allelic variation in genes affecting Process-S as oppose to Process-C.

In both vertebrates and invertebrates, mutations in any of the core clock genes or molecular regulators of the circadian clock have been shown to have drastic effects on period length [3], [4]. Specifically, individuals harboring these mutations have been shown to have very robust activity rhythms that either increase or decrease from the standard 24-hour rhythm. The molecular mechanisms underlying the shift in period length from a 24 hour rhythm to a near 38 hour rhythm in *S. oedipus* remain unclear. Future work on the genetics of circadian rhythms in these populations will prove fruitful to our understanding of how the clock evolves. Importantly, because only one of the 4 cave species studies showed significant changes in period length, but all populations studied showed significantly reduced rest time, we believe that whatever the genetic mechanisms leading to change in rhythmicity may be, they are independent of reduced rest time. A number of studies in both vertebrates and invertebrates suggest that rest time and feeding are tightly interconnected. One hypothesis is that the reduced rest in the cave is advantageous because it increases foraging time in a food poor environment [15], [17], [18]. We believe our data show that reduction in total rest time repeatedly accompanies adaptation to aquatic cave life is, and we believe these data are the first to suggest that loss of total rest occurs independent of circadian Process-C.

## Materials and Methods

### Ethics Statement

All animal work was conducted according to relevant national and international guidelines. This work was approved by the University Animal Welfare Committee of New York University (Protocol numbers 00-1022 and 00-1015). The fish were collected by colleagues in the Thai Departments of Fisheries and Forestry (Chavalit Vidthayanon and Dean Smart); all appropriate collection permits were obtained in advance.

### Animals

All animals were housed in our fish facility in 17L aquaria with water temperature maintained at 21°C±1°C. The individuals were maintained on a diet of fish flakes (TetraMin, fed once a day. All tanks were supplemented with an aerator to maintain air saturated oxygen levels and fish were maintained on a 12∶12 Light∶Dark cycle.

A total of seven balitorid species, two surface dwelling forms and five cave dwelling forms, were used in the present study. All fish except for *Schistura kaysonei* were collected on field trips in 1997, 1999, 2003. Two surface species, *S. mahnerti* and *S. similis* (n = 4), were collected in Mae Hong Son and Tak Provinces , Thailand. Four cave species native to Thailand were collected: *S. jaruthanini* (Tham Sao Hin Cave, Lam Khlong Ngu National Park, Kanchanaburi Province; n = 5) and *Nemacheilus troglocataractus* (Tham Wang Badan Cave, Erawan National Park, Kanchanaburi Province; n = 2), *S. oedipus* (Tham Mae Lana Cave, Mae Hong Son Province; n = 5) and *S. spiesi* (Tham Phra Wang Daeng Cave, Thung Salaeng Luang National Park, Phitsanoluk Province; n = 2). One other cave dwelling species, *S. kaysonei*, native to the Khammouan Karst in central Laos (Phu Tham Nam cave, Ban Don Yom in Khammouane Province; n = 11) was obtained commercially.

### Behavioral Analysis

Locomotor activity was assessed using an automated tracking system similar to those previously described [15], [19]. Individual fish were housed in 17L aquaria and maintained in a laboratory setting on a 12∶12 LD cycle for no less than six months prior to recording. Constant Dark recordings were carried out in standard 17L aquaria. Because water circulation and aeration were essential to the health of the fish, each tank was equipped with an external circulating filter; a nylon wool bridge from the lip of the filter to the edge of the water was used to minimize rippling. Fish were moved from the laboratory aquaria to the test aquaria in a constant dark room and given two days to acclimate to their settings. We then recorded locomotor activity following the two-day acclimation period.

To monitor locomotor activity, a High Resolution Black and White CCD Camera (model number BL58D; ISO Rainbow, Costa Mesa, CA) fitted with a fixed 8 mm focal length lens (model number L8CSWI; ISO Rainbow, Costa Mesa, CA) was mounted above the tanks. Recordings were carried out for a period of 5–8 days using Ethovision software (Noldus Information Technology, Leesburg, VA). Recorded tracks were subsequently analyzed in 1 min bins for average velocity, total distance and movement per second.

To establish locomotor activity in L∶D conditions, fish were also maintained for a period greater than six months in standard 17L aquaria on a 12∶12 L∶D cycle. On the night before the recordings, fish were transferred to a recording chamber that was continuously illuminated with a custom built Infrared (IR; 940 nm) LED source (Part Number 106526; Jameco Electronics, Belmount, CA) and a 12∶12 white light source (175 lux). The timing of the white light corresponded to the entrainment period. The tanks were placed in a recording chamber at least 18 hours prior to recording.

### Analysis and Statistics

Circadian rhythms were analyzed using the Lomb-Scargle algorithm calculated with PEANUTS software [20]. Rest-Activity patterns were analyzed using custom written MatLab scripts as previously described [15]. A Rest bout was scored as any period of complete inactivity lasting at least 10-minutes. Using this criterion, we calculated total rest, daytime rest, nighttime rest, total number of daytime rest bouts, total number of nighttime rest bouts, average daytime rest bout duration, average nighttime rest bout duration, total activity per minute and activity per waking minute.

Statistical testing was done using non-parametric methods implemented in Statistica v9.1 (Stat-Soft, Tulsa, OK). The Kruskal Wallis nonparametric analysis of variance (ANOVA) was used to test significant differences among groups. When significance among groups existed, the ANOVA was followed by post-hoc comparisons among groups with statistical correction for the number of comparisons made. Graphs were made using either Statistica (Stat-Soft, Tulsa, OK) or Graphpad Prism v5 (Graphpad, La Jolla, CA).
